# Emotion Recognition Using Hierarchical Spatiotemporal Electroencephalogram Information from Local to Global Brain Regions

**DOI:** 10.3390/bioengineering10091040

**Published:** 2023-09-04

**Authors:** Dong-Ki Jeong, Hyoung-Gook Kim, Jin-Young Kim

**Affiliations:** 1 Department of Electronic Convergence Engineering, Kwangwoon University, 20 Gwangun-ro, Nowon-gu, Seoul 01897, Republic of Korea; jdklist@kw.ac.kr; 2 Department of ICT Convergence System Engineering, Chonnam National University, 77 Yongbong-ro, Buk-gu, Gwangju 61186, Republic of Korea; beyondi@jnu.ac.kr

**Keywords:** emotion recognition, electroencephalography, hierarchical spatiotemporal features, self-attention, bidirectional gated recurrent unit

## Abstract

To understand human emotional states, local activities in various regions of the cerebral cortex and the interactions among different brain regions must be considered. This paper proposes a hierarchical emotional context feature learning model that improves multichannel electroencephalography (EEG)-based emotion recognition by learning spatiotemporal EEG features from a local brain region to a global brain region. The proposed method comprises a regional brain-level encoding module, a global brain-level encoding module, and a classifier. First, multichannel EEG signals grouped into nine regions based on the functional role of the brain are input into a regional brain-level encoding module to learn local spatiotemporal information. Subsequently, the global brain-level encoding module improved emotional classification performance by integrating local spatiotemporal information from various brain regions to learn the global context features of brain regions related to emotions. Next, we applied a two-layer bidirectional gated recurrent unit (BGRU) with self-attention to the regional brain-level module and a one-layer BGRU with self-attention to the global brain-level module. Experiments were conducted using three datasets to evaluate the EEG-based emotion recognition performance of the proposed method. The results proved that the proposed method achieves superior performance by reflecting the characteristics of multichannel EEG signals better than state-of-the-art methods.

## 1. Introduction

Emotional recognition is considered an important topic in various fields, such as human–computer interaction, psychological research, and neuroscience [1,2,3,4]. Emotions are crucial in daily life and significantly influence behavior, communication, thinking, and mental health [5]. Thus, if machines can recognize human emotions, they offer innovative potential in various applications, including designing more effective computer systems, providing personalized services, and diagnosing and treating mental health [6].

In early emotion research studies, numerous attempts were made to recognize emotions using external signals, such as facial expressions, voices, and behavioral patterns [7,8,9,10]. However, these external signals can be judged differently depending on the subjective interpretation, and discrepancies may arise between outwardly expressed and actual inner emotions [11]. For this reason, with the development of noninvasive sensor technology, the research and application of emotion recognition using biosignals has recently attracted considerable attention [12]. Biological signals [13,14] measure a person’s physical condition, nervous system activity, and physiological responses, including electrocardiograms, skin conductivity, electromyography, and electroencephalography (EEG). Such biometric signals may reflect changes in the internal state of a person and provide information related to emotions and emotional reactions [15].

Among these biosignals, EEG, which is measured from the brain and is directly involved in the processing, generation, and control of emotions, is considered a crucial biosignal for emotion recognition and has several advantages over other modalities. EEG can directly evaluate brain areas related to emotions by directly measuring electrical activities in the brain, provides high temporal resolution to detect and analyze changes in emotions quickly, and can be applied to medical and clinical settings because it detects a wide range of emotions. In addition, emotion recognition using EEG is useful for personalized emotion recognition models and real-time emotion monitoring. Therefore, emotion recognition using EEG can understand and interpret an individual’s internal state and emotions more accurately [16].

Initially, single-channel EEG was primarily used to record brain waves using a single electrode [17]. However, the need for multichannel EEG recordings to analyze the activities occurring in various brain regions has gradually emerged, and now multichannel EEG systems that record brain waves simultaneously using dozens of electrodes are widely used. In emotion recognition studies, multichannel EEG can be used to understand the brain’s emotional processing mechanisms and develop more accurate and comprehensive emotion recognition systems by providing local activities associated with specific brain regions, network interactions among various brain regions, and individual differences and changes in emotions [18].

Deep learning technology, which has rapidly been developing and becoming commonplace in recent years, has achieved remarkable results in EEG-based emotion recognition, surpassing traditional machine learning methods [19,20,21,22]. Li et al. [23] used a hierarchical convolutional neural network (CNN) for EEG-based emotion classification to hierarchically extract features contained in the spatial topology of electrodes that are neglected in a one-dimensional deep model, such as a stacked autoencoder. Li et al. [24] improved the emotion recognition performance using multichannel EEG signals by constructing a hybrid deep learning model that combined CNNs and recurrent neural networks (RNNs). The proposed model effectively captured the interchannel correlation and contextual information. Chen et al. [25] proposed a hierarchical BGRU network with attention mechanism. This model reflects the hierarchical structure of EEG signals and learns important features by utilizing the attention mechanism at both the sample and epoch levels.

However, such existing approaches still have some limitations. First, the research results of EEG-based emotion recognition using deep learning methods still lag behind those of image and speech recognition. Therefore, to develop a more accurate and reliable emotion recognition model, an improved deep learning model that reflects EEG characteristics is required. Second, according to neurological studies, human emotions are closely related to various areas of the cerebral cortex, such as the amygdala, frontal lobe, and parietal lobe [26,27], and spatiotemporal information from different brain regions helps us understand human emotions [28,29,30]. However, these neurological research results are not sufficiently reflected. Because the contribution of EEG signals related to each brain region is different, a method that can utilize the spatiotemporal information of different brain regions is required to understand human emotional states. Third, information about spatial resolution, brain region separation, brain network analysis, individual differences in emotions, and capturing dynamic changes in emotions, which multichannel EEG includes, is not fully utilized. To this end, new studies have recently attempted to utilize the advantages of multichannel EEG fully to identify activities in specific regions of the brain and perform more accurate and multifaceted emotion recognition through the interaction and information combination among brain regions. However, the number of studies is extremely small. Zhang et al. [31] used a hierarchical spatiotemporal EEG feature learning model with attention-based antagonism neural network, whereas Wang et al. [32] proposed a transformer-based model to hierarchically learn the discriminative spatial information from the electrode level to the brain-region level.

Motivated by these research results and limitations, a more accurate and reliable emotion recognition model was developed by reflecting EEG characteristics based on neurological studies. In this paper, we propose an EEG-based emotion recognition method using a hierarchical spatiotemporal context feature learning model (HSCFLM) that extracts local and global EEG features by robustly learning the spatiotemporal dependencies within and among brain regions.

- The main contributions of this paper are:
- To understand the activity of certain brain regions based on human emotions and to enable the interaction among brain regions and the combination of information, we propose a hierarchical neural network model structure with self-attention. The hierarchical deep neural network model with self-attention comprises a regional brain-level encoding module and a global brain-level encoding module;
- To obtain activity weights for each brain region according to emotional state, a regional brain-level encoding module was designed based on a dual-stream parallel double BGRU encoder with self-attention (2BGRUA) for extracting spatiotemporal EEG features. In particular, the spatial encoder learns the interchannel correlations and the temporal encoder captures the temporal dependencies of the time sequence of the EEG channels. Subsequently, the output of each encoder can be integrated to obtain the local spatiotemporal EEG features;
- Next, the global spatiotemporal encoding module uses a single BGRU-based self-attention (BGRUA) to integrate important information within various brain regions to improve the emotion classification performance by learning discriminative spatiotemporal EEG features from local brain regions to the entire brain region. Thus, the influence of brain regions with a high contribution is strengthened by the learned weights, and the influence of the less dependent regions is reduced.

The remainder of this paper is organized as follows: Section 2 introduces the proposed method, Section 3 describes the experimental data and results, and Section 4 presents the conclusions of the study and future research directions.

## 2. Proposed Hierarchical Spatiotemporal Context Feature Learning Model for EEG-Based Emotion Recognition

**Figure 1:**
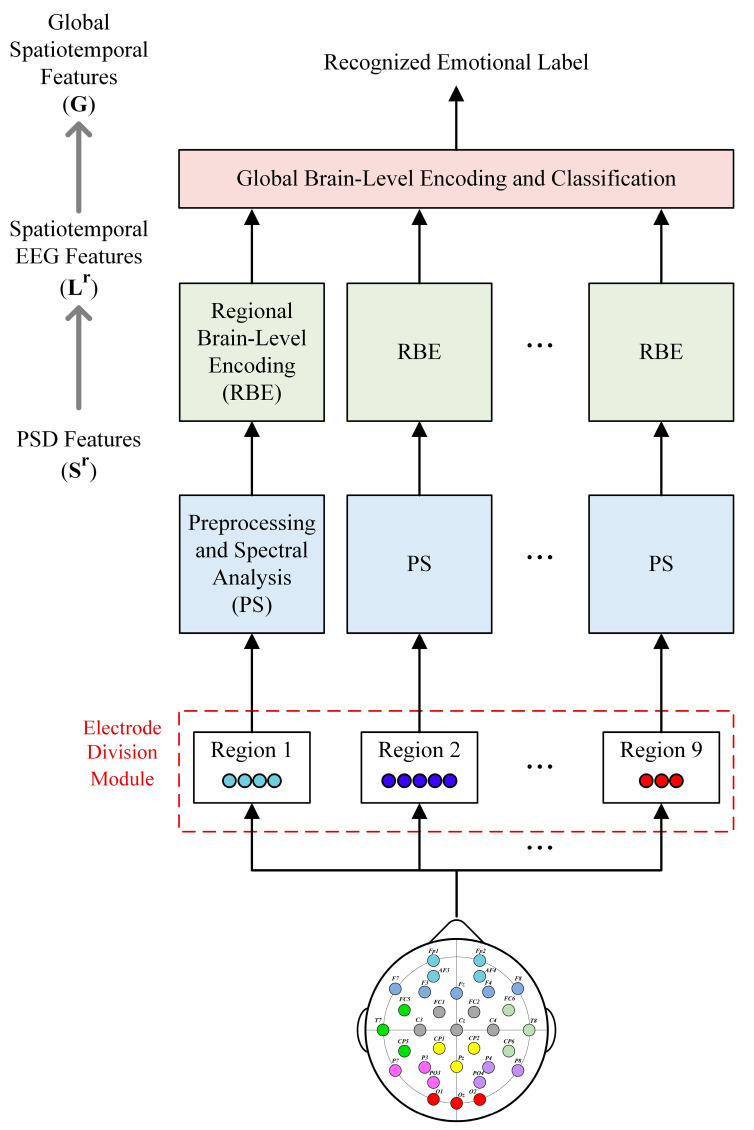
Block diagram of the proposed HSCFLM.

The proposed method for EEG-based emotion classification is a hierarchical neural network architecture with self-attention called the HSCFLM, which mainly consists of three key modules: electrode division module, regional brain-level encoding module, and global brain-level encoding and classification module, as shown in Figure 1. First, EEG channels are grouped by brain region according to the spatial location of the electrodes, because each brain region has a different function. Each grouped regional EEG signal is preprocessed and inputted into the regional brain-level encoding module to extract the spatiotemporal features of each region. After learning the regional deep features, the global brain-level encoding module is used to learn the global emotional EEG context features from local to global brain regions, which are then input into the emotion classifier. The remainder of this section introduces these three key modules.

### 2.1. Electrode Division

**Figure 2:**
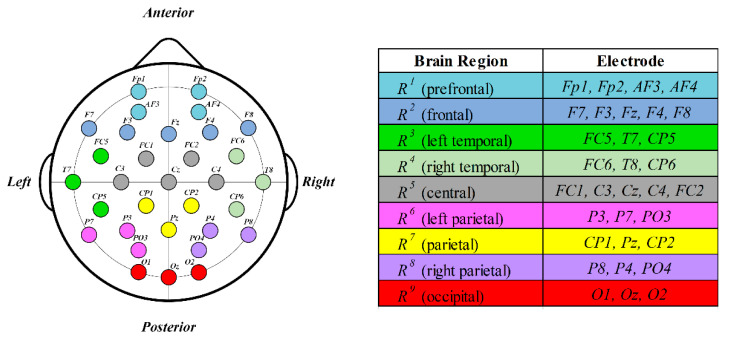
Example of electrode division by brain region (for 32-channel EEG electrode placement based on the international 10–20 system).

The human brain consists of several regions, each performing its own function. These include the frontal, temporal, central, parietal, and occipital regions. Each of these regions shows different brain activity depending on the emotion, which is crucial in understanding complex human emotions. Applying this phenomenon to analyze the correlation among brain regions that respond to human emotional changes, we divided these regions into nine regions based on structure and function [33]: prefrontal, frontal, left temporal, right temporal, central, left parietal, parietal, right parietal, and occipital. Based on the nine brain regions classified above, each electrode can be divided into the corresponding brain region groups based on the position of the electrode attached to the brain to measure the multichannel EEG signal. An example is shown in Figure 2. Electrodes, divided by region, are shown on the left side of Figure 2, and electrodes that belong to the same region are marked with the same color. This information is summarized in the table on the right-hand side. Each region of the brain is expressed as $R^{r}\;\left(r=1,2,\ldots ,9\right)$, and electrodes *Fp1*, *Fp2*, *AF3*, and *AF4* are assigned to $R^{1}$ (prefrontal); *F7*, *F3*, Fz, F4, and F8 to $R^{2}$ (frontal); *FC5*, *T7*, and *CP5* to $R^{3}$ (left temporal); *FC6*, *T8*, and *CP6* to $R^{4}$ (right temporal); FC1, C3, Cz, C4, and FC2 to $R^{5}$ (central); *P3*, *P7*, and *PO3* to $R^{6}$ (left parietal); *CP1*, *Pz*, and *CP2* to $R^{7}$ (parietal); *P8*, *P4*, and *PO4* to $R^{8}$ (right parietal); and *O1*, *Oz*, and *O2* are assigned to $R^{9}$ (occipital).

### 2.2. Preprocessing and Spectral Analysis

Although there may be differences depending on the recording conditions and devices, in general, EEG signals are recorded at an extremely low-voltage level of approximately 1 to 100 μV and include various noise signals in addition to signals generated by the brain itself. Specifically, artifacts from eye blinking, which typically have a higher voltage level than normal EEG signals from emotional stimuli, can lead to a loss of emotion-related EEG signals; therefore, they must be removed to improve the accuracy of emotion recognition.

The preprocessing and spectral analysis are performed on the EEG signals for each brain region. The preprocessing consists of bandpass filtering and downsampling, removal of eye-blink artifacts, and segmentation.

First, a bandpass filter of 4–47 Hz is applied to the EEG signal, and downsampling was performed to 128 Hz. This removes background noise by maintaining only signals in the 4–47 Hz band, eliminating the rest, and reducing the amount of EEG data through downsampling.

Second, eye-blink artifacts are removed from the EEG signal using an optimally modified log-spectral amplitude speech estimator and a minima controlled recursive averaging noise estimation (OM-LSA)-based algorithm [34]. This process comprised the following steps—(Step 1) Artifact detection: Using the OM-LSA-based algorithm, the positions of eye-blink artifacts in the EEG signal are detected. In this process, the start and end points of the artifact are found, defining this as the artifact occurrence section (AOS). (Step 2) AOS length calculation: The number of sample data points in the AOS is calculated using information from its start and endpoints. (Step 3) Acquisition of sample data: Previous samples of the AOS length from the AOS start point and subsequent samples of the AOS length from the AOS endpoint are acquired. (Step 4) Overlap-and-add operation [35]: An operation is performed to overlap and add the EEG samples of the two sections acquired in the previous step. Thus, signals similar to EEG signals related to real emotions are generated. (Step 5) Data replacement and loss concealment: The eye-blink artifact located in the AOS is replaced with the EEG signal generated in the previous step. Thus, the eye-blink artifact is removed, and the information loss of the EEG signal is concealed.

Third, the EEG signals for each brain region from which noise and artifacts are removed are divided into 1 s segments, and each segment overlaps by 50%. Each segmented EEG signal is used in the subsequent step of the spectral analysis of each brain region.

The five main frequency bands of the brain waves are delta (1–4 Hz), theta (4–7 Hz), alpha (8–12 Hz), beta (13–30 Hz), and gamma (31–47 Hz), which are associated with different emotional states. By converting these EEG signals into a power spectral density (PSD), different emotional states can be effectively distinguished and recognized through the distribution pattern of the signal power for each band. To this end, each EEG segment is analyzed with a short-time Fourier transform using a 0.25 s sliding window with 80% overlap. Here, the PSD is calculated using the average power in four frequency bands, excluding the delta band related to deep sleep. Thus, the PSD feature expression $S^{r}=\left[p_{t1}^{r},p_{t2}^{r},\ldots ,\;p_{tN_{r}}^{r}\right]\in R^{d\times N_{r}\times T}$ for brain region $R^{\;r}$ is the output. Here, $N_{r}$ and T denote the number of channels and segments in the EEG sequence for the brain region $R^{r}$, respectively. Furthermore, d represents the dimensions of the PSD features extracted from a single channel. Feature vector $S^{r}$ is used as the input for regional brain-level encoding.

### 2.3. Regional Brain-Level Encoding Module

Certain emotions can be associated with specific brain regions. For example, fear-related brain regions are deeply related to the amygdala, and love-related emotions do not focus on just one area of the brain but involve the interaction of several areas of the brain. However, certain brain areas are related to the experience of love and romantic emotions. Simultaneous recordings of brain waves occurring in different brain regions using multichannel EEG can identify local spatiotemporal pattern changes in the brain activity associated with specific emotions.

**Figure 3:**
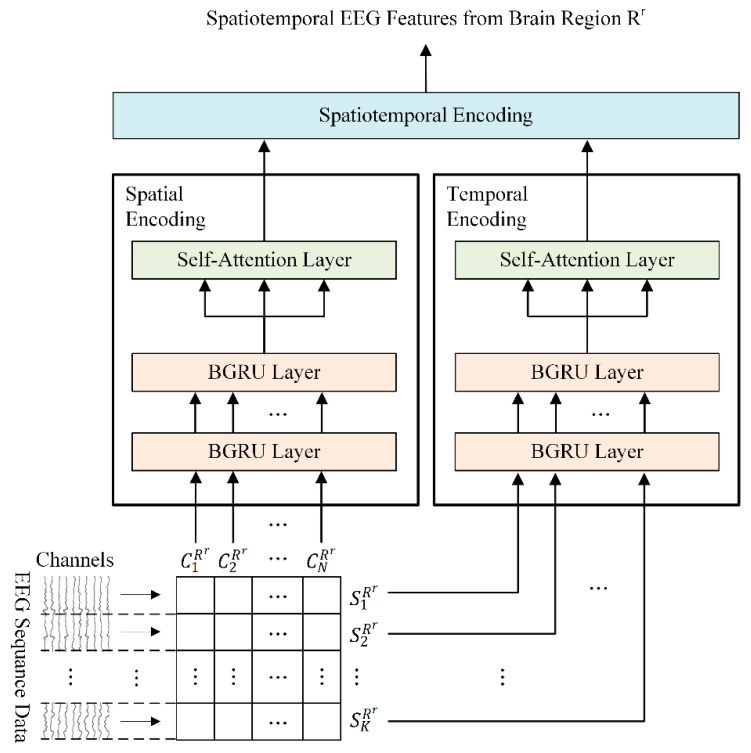
Overview of the regional brain-level encoder in the HSCFLM.

For this reason, we designed a regional brain-level encoding module to effectively learn spatiotemporal EEG feature representations using both the spatial and temporal information of each brain region. The regional brain-level encoding module has a dual-stream parallel structure and consists of two submodules, a spatial encoder, and a temporal encoder, the structure of which is shown in Figure 3.

The PSD feature expression $S^{r}$, obtained through preprocessing and spectrum analysis, is configured as in Equations (1) and (2) and input into spatial and temporal encoders in parallel.

(1) $$H^{r}=\left[c_{1}^{r},c_{2}^{r},\ldots ,\;c_{n}^{r},\ldots ,\;c_{N_{r}}^{r}\right]\in R^{d\times N_{r}}$$

(2) $$U^{r}=\left[s_{1}^{r},s_{2}^{r},\ldots ,s_{t}^{r},\ldots ,\;s_{T}^{r}\right]\in R^{d\times T}$$

where $c_{n}^{r}$ represents the PSD feature vector of the n-th channel of the EEG channel sequence $H^{r}$, and $s_{t}^{r}$ represents the PSD feature vector of the t-th segment of the EEG time sequence $U^{r}$.

Both the spatial and temporal encoders are implemented based on a two-layer BGRU with self-attention (2BGRUA). The spatial encoder utilizes 2BGRUA to learn the interchannel correlations of the input regional EEG channels. We also employed 2BGRUA, as shown in Figure 3, as a temporal encoder to learn the key temporal features of the time-sequential EEG data. The EEG sequences $H^{r}$ and $U^{r}$ are input into the first BGRU layer of the spatial and temporal encoders composed of 2BGRUA. The process up to the second layer of BGRU is performed using Equations (3)–(6).

(3) $$BGRU\left(H^{r}\right)={\overset{ˇ}{H}}^{r}=\left[h_{1}^{C^{r}},h_{2}^{C^{r}},\ldots ,h_{n}^{C^{r}},\ldots ,h_{N_{r}}^{C^{r}}\right]$$

(4) $$BGRU\left(U^{r}\right)={\overset{ˇ}{U}}^{r}=\left[h_{1}^{S^{r}},h_{2}^{S^{r}},\ldots ,h_{t}^{S^{r}},\ldots ,h_{T}^{S^{r}}\right]$$

(5) $$BGRU\left({\overset{ˇ}{H}}^{r}\right)={\hat{H}}^{r}=\left[g_{1}^{C^{r}},g_{2}^{C^{r}},\ldots ,g_{n}^{C^{r}},\ldots ,g_{N_{r}}^{C^{r}}\right]$$

(6) $$BGRU\left({\overset{ˇ}{U}}^{r}\right)={\hat{U}}^{r}=\left[g_{1}^{S^{r}},g_{2}^{S^{r}},\ldots ,g_{t}^{C^{r}},\ldots ,g_{T}^{S^{r}}\right]$$

where $BGRU\left(\cdot \right)$ denotes the BGRU operation; ${\overset{ˇ}{H}}^{r}$ and ${\overset{ˇ}{U}}^{r}$ denote the output vectors of the first BGRU layer for the input sequences $H^{r}$ and $U^{r}$, respectively; and ${\hat{H}}^{r}$ and ${\hat{U}}^{r}$ denote the output vectors of the second BGRU layer. $h_{n}^{C^{r}}\in R^{2d_{hc}}$ and $h_{t}^{S^{r}}\in R^{2d_{hs}}$ represent the hidden state vectors output by the n-th and t-th bidirectional hidden units of the first BGRU layer, respectively. $d_{hc}$ and $d_{hs}$ denote the dimensions of the hidden state vector. Furthermore, $g_{n}^{C^{r}}\in R^{2d_{gc}}$ and $g_{t}^{S^{r}}\in R^{2d_{gs}}$ represent the hidden state vectors output by the n-th and t-th bidirectional hidden units of the second BGRU layer. The dimensions of each hidden unit state vector are $d_{gc}$ and $d_{gs}$, respectively.

**Figure 4:**
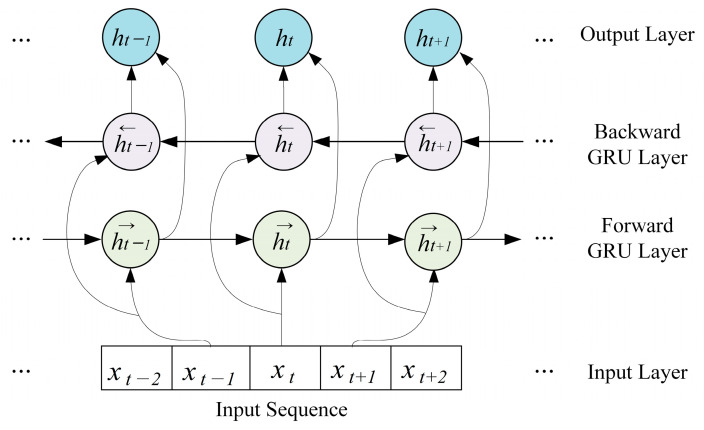
Structure of the bidirectional gated recurrent unit.

Specifically, as shown in Figure 4, the BGRU has a structure in which a hidden layer that processes sequences in reverse order is added to the structure of the GRU [36], which consists of only forward layers that process input sequences sequentially. This structure allows the BGRU to learn long-term dependencies better than the GRU by considering both the past and future states of the input sequence.

The forward and backward context feature representations for an input sequence extracted via BGRU are as follows in (7) and (8):

(7) $${\vec{h}}_{t}={\vec{GRU}}_{f}\left(x_{t}\right),t\in \left[1,T\right]$$

(8) $${\overset{\leftarrow }{h}}_{t}={\overset{\leftarrow }{GRU}}_{b}\left(x_{t}\right),t\in \left[T,1\right]$$

where ${\vec{h}}_{t}$, ${\overset{\leftarrow }{h}}_{t}$, ${\vec{GRU}}_{f}$, ${\overset{\leftarrow }{GRU}}_{b}$, and $x_{t}$ denote the forward hidden sequence, backward hidden sequence, forward GRU, backward GRU, and the feature vector of the sample at time t, respectively. ${\vec{h}}_{t}$ is obtained by processing the input sample sequence in order from time step t=1 to T through ${\vec{GRU}}_{f}$, and ${\overset{\leftarrow }{h}}_{t}$ is obtained by processing the data in reverse order from t=T to 1. The obtained forward and backward context feature vectors ${\vec{h}}_{t}$ and ${\overset{\leftarrow }{h}}_{t}$ are summarized in the bidirectional context feature expression $h_{t}$, as shown in Equation (9), using a concatenation operation:

(9) $$h_{t}=\left[{\vec{h}}_{t},{\overset{\leftarrow }{h}}_{t}\right]$$

**Figure 5:**
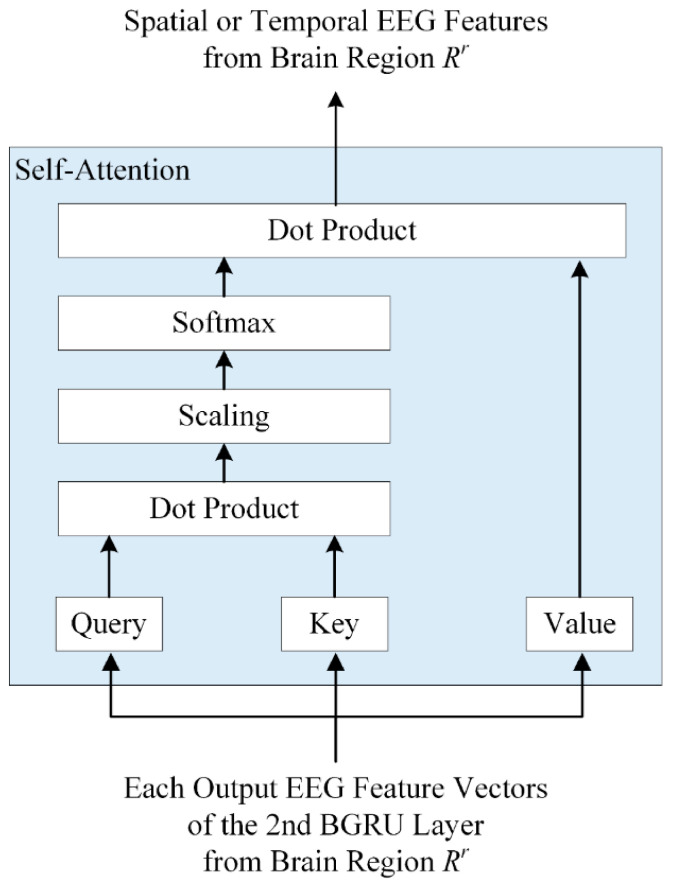
Structure of self-attention layer.

The 2BGRUA model builds two BGRU networks in series and adds a self-attention mechanism. In this hybrid model, the first BGRU layer extracts future and past time series EEG information, and the second BGRU layer is applied to learn more significant feature representation, while better emotional feature information is provided by assigning weights to contextual feature information using a self-attention mechanism. In general, self-attention can effectively model global interactions; however, it has limited disadvantages in figuring out local dependencies. In contrast, the BGRU can effectively identify regional dependencies that reflect bidirectional temporal patterns and flows within the input sequence. Therefore, we applied the BGRU before self-attention. Figure 5 shows the structure of the self-attention layer.

First, each feature vector ${\hat{H}}^{r}$ and ${\hat{U}}^{r}$ output through the second BGRU layer is input into self-attention and is output as each query, key, and value by multiplying with the corresponding weight matrix, as shown in Equations (10) and (11):

(10) $$Q^{C^{r}}={\hat{H}}^{r}W_{Q}^{C^{r}},\;K^{C^{r}}={\hat{H}}^{r}W_{K}^{C^{r}},\;V^{C^{r}}={\hat{H}}^{r}W_{V}^{C^{r}}$$

(11) $$Q^{S^{r}}={\hat{U}}^{r}W_{Q}^{S^{r}},\;K^{S^{r}}={\hat{U}}^{r}W_{K}^{S^{r}},\;V^{S^{r}}={\hat{U}}^{r}W_{V}^{S^{r}}$$

where $Q^{C^{r}}$, $K^{C^{r}}$, and $V^{C^{r}}$ denote the query, key, and value by the linear transformation of input ${\hat{H}}^{r}$, respectively, and $Q^{S^{r}}$, $K^{S^{r}}$, and $V^{S^{r}}$ refer to the query, key, and value by the linear transformation of input ${\hat{U}}^{r}$, respectively. $W_{Q}^{C^{r}}$, $W_{K}^{C^{r}}$, $W_{V}^{C^{r}}$, $W_{Q}^{S^{r}}$, $W_{K}^{S^{r}}$, and $W_{V}^{S^{r}}$ indicate the learnable weight matrices.

Next, the similarity between the query and key vectors is calculated using the dot product. The calculated similarity is divided by the square root of the dimension of the key vector, and the probability distribution is calculated by applying a softmax function to the result to obtain each attention score. A dot product operation is then performed between the obtained attention score and the value vector. Thus, each feature vector, $Attention\left(Q^{C^{r}},K^{C^{r}},V^{C^{r}}\right)$ and $Attention\left(Q^{S^{r}},K^{S^{r}},V^{S^{r}}\right)$ in which the attention weight is reflected, is obtained. These are the output feature representations of the spatial and temporal encoders, respectively, and are expressed by Equations (12) and (13).

(12) $$Attention\left(Q^{C^{r}},K^{C^{r}},V^{C^{r}}\right)=softmax\left(\frac{Q^{C^{r}}{\left(K^{C^{r}}\right)}^{T}}{\sqrt{d_{K^{C^{r}}}}}\right)V^{C^{r}}$$

(13) $$Attention\left(Q^{S^{r}},K^{S^{r}},V^{S^{r}}\right)=softmax\left(\frac{Q^{S^{r}}{\left(K^{S^{r}}\right)}^{T}}{\sqrt{d_{K^{S^{r}}}}}\right)V^{S^{r}}$$

where T represents the matrix transpose, $d_{K^{C^{r}}}$ and $d_{K^{S^{r}}}$ are the dimensions of the key vectors $K^{C^{r}}$ and $K^{S^{r}},$ respectively. softmax(.) and Attention(.) denote the softmax function and self-attention operation, respectively.

Through the previous process, each output feature extracted independently from the spatial and temporal encoders is input into the spatiotemporal encoding module and fused into a spatiotemporal EEG feature $L^{r}$ for the corresponding brain region $R^{r}$ using concatenation operations, as shown in Equation (14).

(14) $$L^{r}=\left[Attention\left(Q^{C^{r}},K^{C^{r}},V^{C^{r}}\right),Attention\left(Q^{S^{r}},K^{S^{r}},V^{S^{r}}\right)\right]$$

Similarly, the local spatiotemporal features extracted from the nine brain regions are all concatenated and output as a local spatiotemporal feature vector L, as shown in Equation (15), which is then used as an input for global brain-level encoding.

(15) $$L=\left[L^{1},L^{2},\ldots ,L^{9}\right]$$

### 2.4. Global Brain-Level Encoding and Classification Module

Emotions are distributed across various brain regions, and each region has temporal and spatial emotional characteristics. The brain is composed of networks, and emotions are associated with interactions among different brain regions. Therefore, emotional recognition performance can be improved by extracting spatiotemporal features from each brain region and learning global spatiotemporal features through interactions among brain regions and combining information. This helps us recognize emotions more accurately by simultaneously considering the activities of various brain regions. The global brain-level encoding model is a key module that can effectively learn spatiotemporal features extracted from various brain regions and improve emotion recognition performance by identifying globally distinct patterns according to various emotional states.

**Figure 6:**
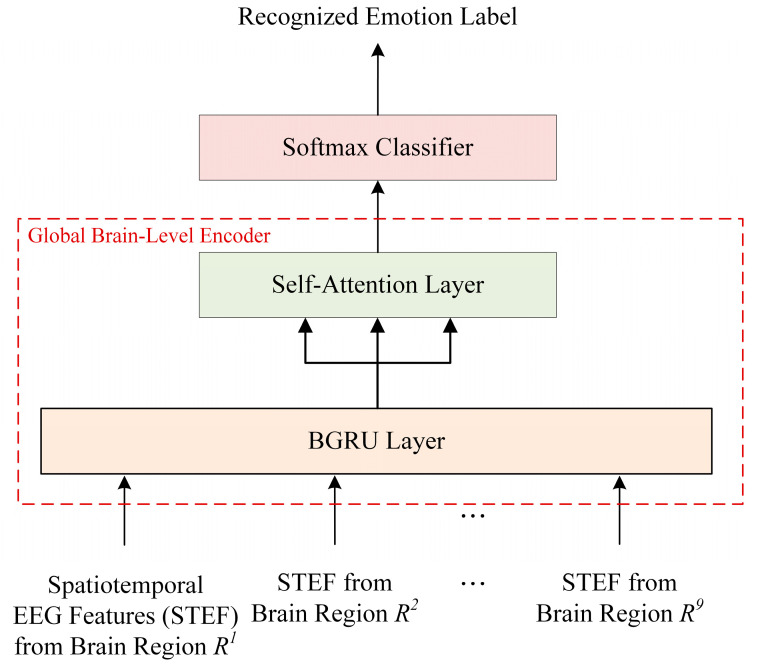
Architecture of the global brain-level encoder and classifier in the HSCFLM.

Figure 6 shows the structure of the global brain-level encoding and classification model, which consists of BGRU, self-attention, and softmax. Unlike the regional brain-level encoder, which uses a two-layer BGRU, the global brain-level encoder uses a single-layer BGRU. This is because it is only used to combine information among brain regions based on spatiotemporal information sufficiently extracted from the regional brain-level.

The local spatiotemporal feature vector L obtained through regional brain-level encoding is input into the BGRU layer, as shown in Equation (16), and the global context feature vector E is obtained.

(16) $$BGRU\left(L\right)=E=\left[h_{1}^{G},h_{2}^{G},\ldots ,h_{9}^{G}\right]\in R^{2d_{g}\times 9}$$

where $d_{g}$ represents the dimension of each hidden unit state vector.

Next, the extracted global context feature vector E is input into the self-attention to extract a global spatiotemporal feature G. This is obtained using Equations (17) and (18):

(17) $$Attention\left(Q^{G},K^{G},V^{G}\right)=softmax\left(\frac{Q^{G}{\left(K^{G}\right)}^{T}}{\sqrt{d_{K^{G}}}}\right)V^{G}$$

(18) $$G=Attention\left(Q^{G},K^{G},V^{G}\right)$$

where $Q^{G}$, $K^{G}$, and $V^{G}$ denote the query, key, and value, respectively, by the linear transformation of the input L. In addition, $d_{K^{G}}$ represents the dimensions of key vector $K^{G}$.

The global spatiotemporal feature G extracted from the global brain-level encoder is input into the softmax classifier for emotional state prediction and used to calculate the probability distribution for the emotion classes.

First, a linear transformation is performed on feature expression G using the learned weight matrix $W_{z}$ and bias vector $b_{z}$, which is expressed in Equation (19):

(19) $$M=W_{z}G+b_{z}=\left[v_{1},\;v_{2},\ldots ,v_{c}\right]$$

where c is the number of emotion categories for classification, and $v_{i}$ represents the raw score for the i-th class.

The obtained raw score M is input into the softmax function to calculate the probability of each emotion class. The classifier outputs the class with the highest probability p as the recognized emotional state label. The calculation for p is as in Equation (20) below.

(20) $$p=max\left\{\frac{exp\left(v_{j}\right)}{\sum_{i=1}^{c}exp\left(v_{i}\right)}\left\lfloor j=1,2,\ldots ,c\right.\right\}$$

Cross-entropy is used as the loss function for model training and is expressed in Equation (21).

(21) $$E=-\frac{1}{Z}\sum_{z-1}^{Z}Y_{z}\log\left({\tilde{Y}}_{z}\left(X_{z},\theta \right)\right)+\frac{\lambda }{2}{\left\|\theta \right\|}_{2}^{2}$$

where $X_{z}$ and $Y_{z}$ denote the z-th EEG sequence and the label of the sequence, respectively. Additionally, θ and λ denote the parameter set and normalization parameters of the model, respectively.

The Adam optimization algorithm is used to train the model to minimize the cross-entropy error between the predicted and actual labels. The weight parameters and biases are adjusted based on the calculated losses. The training process is continued until either the desired performance or the best possible performance was achieved.

## 3. Experiment and Results

The DEAP, MAHNOB-HCI, and SEED datasets were used to evaluate the performance of the proposed method. All three datasets include EEG data generated by audiovisual emotional stimuli.

### 3.1. Evaluation Datasets

The performance of the proposed model was evaluated using three public datasets containing EEG signals generated by audiovisual stimuli.

- DEAP [37]: The DEAP dataset contains EEG and peripheral signals from 32 participants (16 males and 16 females between the ages of 19 and 37 years). EEG signals were recorded while each participant watched 40 music video clips. Each participant watched each video and rated the levels of arousal, valence, dominance, and liking on a continuous scale of 1 to 9 using a self-assessment manikin (SAM). Each trial contained a 63 s EEG signal. The first 3 s of the signal is the baseline signal. EEG signals were recorded at a sampling rate of 512 Hz using 32 electrodes. In this paper, EEG data from 24 participants (12 males and 12 females) were selected for the experiment;
- MAHNOB-HCI [38]: The MAHNOB-HCI dataset includes the EEG and peripheral signals. EEG signals were collected at a sampling rate of 256 Hz from 32 electrodes while each of the 27 participants (11 males and 16 females) watched 20 selected videos. The video clip used as the stimulus had a length of approximately 34–117 s. Each participant watched each video and self-reported the levels of arousal, valence, dominance, and predictability through the SAM on a nine-point discrete scale. For the various experiments performed in this study, 22 of the 27 participants (11 male and 11 female) were selected;
- SEED [39]: The SEED dataset provides EEG and eye-movement signals from 15 participants (7 males and 8 females). The EEG signals of each participant were collected while watching 15 Chinese movie clips, each approximately four minutes long, designed to elicit positive, neutral, and negative emotions. The sampling rate of the signal collected using the 62 electrodes was 1 kHz, which was later downsampled to 200 Hz. After watching each movie clip, each participant recorded the emotional label for each video as negative (−1), neutral (0), or positive (1). The experiment was performed using EEG data from 12 of the 15 participants (6 males and 6 females) were used.

### 3.2. Experimental Methods

To evaluate the emotion classification performance of the proposed method and compare it with other neural network models, the various methods listed below were applied in the experiment.

- CNN: This method constituted two convolution layers with four 3 × 3 convolutional filters, two max-pooling layers with a 2 × 2 filter size, a dropout layer, two fully connected layers, and a softmax layer;
- LSTM: LSTM was applied rather than a CNN. It comprised two LSTM layers: a dropout, fully connected and a softmax layers. The number of hidden units of the lower- and upper-layer LSTMs were 128 and 64, respectively. The fully connected layer contained 128 hidden units;
- BGRU: BGRU was used rather than LSTM. The number of hidden units in the forward and backward GRU layers was 64;
- Convolutional recurrent neural network (CRNN) [24]: A hybrid neural network consisting of a CNN and an RNN for extracting spatiotemporal features was applied to multichannel EEG sequences. This network consisted of two convolution layers, a subsampling layer, two fully connected recurrent layers, and an output layer;
- Hierarchical-attention-based BGRU (HA-BGRU) [25]: The HA-BGRU consisted of two layers. The first layer encoded the local correlation between samples in each epoch of the EEG signal, and the second layer encoded the temporal correlation between epochs in an EEG sequence. The BGRU network and attention mechanism were applied at both sample and epoch levels;
- Region-to-global HA-BGRU (R2G HA-BGRU): the HA-BGRU network was applied to extract regional features within each brain region and global features between regions;
- R2G transformer encoder (TF-Encoder): in the R2G HA-BGRU method, transformer encoders were applied instead of BGRU with attention mechanism;
- R2G hierarchical spatial attention-based BGRU (HSA-BGRU): only temporal encoding in a regional brain-level encoding module was used, and the same method as the HSCFLM was applied for the rest;
- HSCFLM: This method mainly consisted of a regional brain-level encoding module, a global brain-level encoding, and classification module. This is the proposed method.

The CNN, LSTM, BGRU, CRNN, and HA-BGRU methods apply multichannel EEG signals to each neural network without dividing the brain into regions. However, the R2G HA-BGRU, R2GTF, R2G HAS-BGRU, and proposed HSCFLM methods divided the brain into nine defined regions, grouping the corresponding electrodes by region. Among them, a soft attention mechanism was applied to the R2G HA-BGRU method, while a self-attention mechanism was applied to the other methods.

Accuracy was used to evaluate the performance of each method and is defined by Equation (22):

(22) $$Accuracy=\frac{TP+TN}{TP+TN+FP+FN}$$

where true positive (TP) refers to the number of positive data points correctly classified as positive. True negative (TN) refers to the number of negative data points correctly classified as negative. False positive (FP) refers to the number of negative data points incorrectly classified as positive. False negative (FN) refers to the number of positive data points incorrectly classified as negative. The accuracy of the model is defined as the ratio of correctly classified data points to the total number of data points.

### 3.3. Experimental Results

For the various experiments evaluating the performance of the proposed model, as shown in Table 1 and Table 2, the labels of the DEAP and MAHNOB-HCI datasets were redefined for several levels of emotion classes based on a rating value of 1–9 in terms of valence, arousal, and dominance. Additionally, by combining each of the two classes for valence and arousal presented in Table 1, the classes for the four-level emotion classification were defined as follows: high valence and high arousal (HVHA), low valence and high arousal (LVHA), low valence and low arousal (LVLA), and high valence and low arousal (HVLA). Because the SEED dataset is labeled as negative (−1), neutral (0), or positive (1), it was only applied to the three-level emotion classification experiment in terms of valence without relabeling.

Table 3, Table 4 and Table 5 show the results of two to four levels of emotion classification using the three datasets. All emotion classification results in Table 3, Table 4 and Table 5 were calculated using leave-one-subject-out (LOSO) cross-validation evaluation [40]. This evaluation method sets the EEG data of one subject as a test set and uses the EEG data of all the remaining subjects as a training set. This process was repeated until each participant’s data were used as a test set at least once. That is, the test and training data were always selected from different subjects. This approach can be used to evaluate the generalizability of the proposed model to new subjects by learning general patterns. The final classification performance results were obtained by calculating the average of all the cross-validation folds.

First, as shown in Table 3, Table 4 and Table 5, the accuracy of the emotion classification gradually decreased as the number of classes increased from two to four in terms of valence, arousal, and dominance in the DEAP and MAHNOB-HCI datasets. This is because as the number of emotion classes to be distinguished increases, the number and complexity of patterns that the model needs to learn increases.

For the two- and three-level emotion classifications using the DEAP dataset, as shown in Table 3, the classification accuracy was high in the order of dominance, valence, and arousal for most of the methods. Alternatively, it exhibited the lowest classification performance in terms of arousal. Similarly, two- and three-level classification using the MAHNOBHCI dataset, as presented in Table 4, showed the same tendency with the lowest classification performance in terms of arousal. This suggests that arousal may represent more complex EEG patterns than either dominance or valence. Additionally, the three-level emotion classification exhibited the highest performance in the SEED dataset among the three datasets. This may be related to the SEED dataset, which employs more spatiotemporal information from numerous local brain regions and more electrodes than the DEAP and MAHNOB-HCI datasets.

In all subject-independent experiments, the proposed method achieved the highest emotion classification accuracy. Table 6 shows the number of learnable parameters to obtain various feature vectors in the application of the proposed HSCFLM. During simulation by connecting the modules of the proposed method, the slopes of all parameters were calculated and optimized using the cross-entropy loss. The obtained optimal parameters were applied to the proposed architecture to provide the highest classification performance. In addition, Table 7 shows the network parameters, training time, test time per EEG data, network size, and average recognition accuracy performance. This represents the complexity of the model from a quantitative perspective. This information was obtained from hardware environments using Windows 10, Intel Core i5 8500 processor, NVIDIA GeForce GTX 1070 Ti GPU, DDR4 32GB RAM, and software environments using TensorFlow 2.9.1 and Python 3.10.8.

As shown in Table 3, Table 4 and Table 5, the R2G HA-BGRU, R2G TF-Encoder, R2G HSA-BGRU, and the proposed HSCFLM method exhibited superior performance in all types of experiments on the DEAP, MAHNOB-HCI, and SEED datasets. This indicates that learning spatiotemporal dependencies within and among brain regions by dividing the brain into areas is more effective for recognizing emotions than extracting temporal and spatial features from multichannel EEG signals without dividing the brain into regions.

Evidently, the R2G HSA-BGRU and HSCFLM methods, which utilize a hybrid structure combining BGRU and self-attention, present superior performance compared to the R2G TF-Encoder method. This suggests that the hybrid structure can more accurately capture regional and global dependencies from EEG signals to extract high-level global EEG features. Furthermore, upon comparing the performance of the R2G HSA-BGRU and HSCFLM methods, it can be observed that the method that considers both temporal and spatial information improves the accuracy of emotion recognition compared with the method that extracts only temporal information. This approach considers brain activity patterns and complex interactions to better understand and reflect complex patterns of emotions.

As a further experiment, we obtained a classification accuracy of 79.1% on the DEAP dataset by dividing the brain region into four primary regions (frontal, parietal, temporal, and occipital lobes) and measuring the performance of the four-level classification of the proposed model. This result was approximately 3.4% lower than the result in Table 4 obtained by dividing the brain into nine regions. Based on this result, it was found that the analysis of various brain regions can improve the accuracy of emotional classification.

To evaluate the performance of the proposed model, a within-subject experiments on emotion classification was also conducted, and the results of these experiments are presented in Table 8. Table 8 contains the results of four-level emotion classification experiments using the DEAP and MAHNOB-HCI datasets and three-level emotion classification experiments using the SEED dataset. In addition, a paired t-test was performed for the proposed method and other neural network models to evaluate the statistical difference in emotion classification performance. EEG data for 10, 8, and 5 subjects in the DEAP, MAHNOB-HCI, and SEED datasets, respectively, were applied. A *p*-value less than 0.05 indicates that the difference is statistically significant. From the experimental results, the average accuracy performance of the proposed model was 92.4%, 93.1%, and 97.2% for the DEAP, MAHNOB-HCI, and SEED datasets, respectively, showing average classification accuracy performance that was statistically significantly higher than those of the other methods.

Performance evaluation of the brain region, based on the emotional state, was performed using the proposed model, and the results are presented in Table 9. The results indicate that emotions can be recognized similarly in all brain regions. More specifically, in the DEAP database the prefrontal lobe (PF), frontal lobe (F), and parietal lobe (P) achieved better performance for arousal classification, which indicates the degree of emotional activation. In contrast, in the MAHNOB-HCI database, we demonstrated that PF, P, and the right parietal achieved better performance. It is estimated that the PF and F are located in the front of the brain and are connected to numerous brain regions that process emotion-related information; therefore, they are crucial to processing and regulating emotion-related information. These results were similar to the SEED database and were consistent with observations in neuroscience. Furthermore, PF, F, left temporal lobe, and right temporal lobe demonstrated superior performance in valence classification, which represents emotional tendencies or moods, across the DEAP, MAHNOB-HCI, and SEED databases. This suggests that the prefrontal, frontal, and temporal lobe contribute more to the valence classification based on emotional experiences and learning.

## 4. Conclusions, Limitations, and Future Work

In this paper, we propose an emotion classification method called the hierarchical emotion context feature learning model, which learns spatiotemporal emotion information from a local brain region to a global brain region. Our method extracts spatiotemporal EEG features that represent activity weights in each brain region in relation to emotions and utilizes a global EEG representation that incorporates correlations in various brain regions. Specifically, we extracted EEG spatiotemporal features using regional brain-level encoders. Using global spatiotemporal encoders, we learned discriminative spatiotemporal EEG features from local brain regions to entire brain regions. Comparative experiments demonstrated the superiority of the proposed method over EEG-based emotion-recognition neural models. Analyzing emotions by extracting regional and global EEG characteristics by learning the spatiotemporal dependence among brain regions through multichannel brainwave signals is a novel task and a successful attempt at emotional analysis research in emotional computing. Thus, we identified certain limitations of the proposed method and identified certain directions for future improvements.

First, the principles and learning processes of the proposed model were clarified, achieving higher classification accuracy compared to conventional methods; however, the model is complex, making it difficult to explain the intermediate decision making and overall interaction. To this end, we intend to increase the possibility of explanations by visualizing internal actions, such as the correlation and interaction among input data, learned features, and output classes. Additionally, we plan to explore methods to enhance the robustness and domain adaptability of the model. This will involve simplifying the model’s structure and parameters and reducing the complexity of the hierarchical deep-learning modules through visual verification.

Second, the experimental results tend to be lower than those achieved in the field of image and speech recognition. This suggests that the imbalance between the volume of EEG data and the complexity of the model does not yield optimal performance in accurately identifying each emotion category. We will solve this problem by extending the EEG data to ensure a balanced database. Along with this, we will aim to adjust and improve the loss function to identify class samples by adding penalty coefficients to the misclassified class samples.

Third, the EEG signals of DEAP and MAHNOB-HCI were recorded using 32 electrodes according to the international 10–20 system, whereas 62 electrodes were used to record the EEG of the SEED. Because the EEG equipment requires a strict laboratory environment, it is difficult to apply EEG-based emotion analysis in practice. Recently, to overcome this problem, portable and reliable EEG devices have been developed, which increases the likelihood of conducting large-scale experiments in real-world applications. Because the number of electrodes in portable EEG devices is relatively small, we intend to contribute to portable EEG devices that can be used in daily life by expanding our future work to explore the location of electrodes to efficiently recognize emotions using our model.

Fourth, only a few experiments were conducted for two, three, and four emotional classes in this study. Therefore, various emotional classes must be captured by introducing additional emotional classes. Thus far, we can add an emotion class by extending Russell’s emotional classification axis from two dimensions to multiple dimensions and extend a more granular emotion class using a classification method with a hierarchical structure. Furthermore, an emotional continuum representing a continuous emotional state can be generated, and various emotional classes can be defined and applied based on this continuum. In future studies, we will carefully select these principles and apply them to regression methods that can learn the relationship between the input variables and the emotional levels necessary for recognizing various emotional classes.

Fifth, in this study, brain region was divided into nine regions and applied to emotion recognition. Dividing the brain region into nine regions reduces the number of electrodes assigned to each region. As a result, the signal of each region may be more concise, but detailed information may be omitted. However, it has the advantage of being able to investigate the relationship between the region and emotions in more detail. On the other hand, if the brain region is divided into four main regions, more electrodes are assigned to each region. This makes it possible to analyze signals in each area in more detail, but it can complicate the analysis task. However, it is useful understand the possibility of generalization of what type of emotion each major area is associated with. For this reason, we will apply the proposed model to study which brain regional division method is most effective for emotion recognition.

## Figures and Tables

**Table 1:** Emotion classes for two-level emotion classification on the DEAP and MAHNOB-HCI datasets.

Rating Values (RVs)	Valence	Arousal	Dominance
1≤RVs≤5	Low	Low	Low
6≤RVs≤9	High	High	High

**Table 2:** Emotion classes for three-level emotion classification on the DEAP and MAHNOB-HCI datasets.

Rating Values (RVs)	Valence	Arousal	Dominance
1≤RVs≤3	Negative	Activated	Controlled
4≤RVs≤6	Neutral	Moderate	Moderate
7≤RVs≤9	Positive	Deactivated	Overpowered

**Table 3:** Experimental results of subject-independent emotion classification using the DEAP dataset.

Methods	Two-Level CL			Three-Level CL		
	VAL	ARO	DOM	VAL	ARO	DOM
CNN	69.7 (11.82)	66.7 (9.50)	70.1 (11.84)	65.3 (10.09)	64.7 (10.43)	65.9 (8.94)
LSTM	75.2 (11.56)	72.3 (10.30)	75.3 (9.11)	71.1 (8.89)	69.7 (11.13)	71.6 (9.46)
BGRU	76.8 (11.62)	74.4 (11.45)	77.2 (8.96)	73.2 (9.02)	71.5 (8.60)	73.1 (8.81)
CRNN [24]	81.1 (9.21)	78.9 (11.31)	81.4 (11.01)	77.4 (11.04)	76.1 (10.43)	77.4 (10.58)
HA-BGRU [25]	83.4 (10.26)	81.5 (10.04)	84.1 (9.42)	80.2 (9.48)	78.8 (10.12)	80.1 (11.34)
R2G HA-BGRU	87.6 (9.73)	85.3 (9.46)	88.1 (11.66)	83.9 (10.54)	82.9 (11.14)	84.1 (10.92)
R2G TF-Encoder	88.1 (10.05)	86.4 (8.37)	88.4 (9.55)	84.5 (10.95)	83.5 (11.29)	84.5 (11.14)
R2G HSA-BGRU	88.4 (8.95)	87.1 (10.79)	89.3 (10.59)	85.1 (9.31)	84.2 (10.43)	85.2 (11.20)
**HSCFLM**	**92.1** **(9.16)**	**90.5** **(9.81)**	**92.3** **(8.94)**	**88.5** **(8.52)**	**87.4** **(8.35)**	**88.3** **(9.76)**

The standard deviation is provided in parentheses. CL, VAL, ARO, and DOM denote classification, valence, arousal, and dominance, respectively.

**Table 4:** Experimental results of subject-independent emotion classification using the MAHNOB-HCI dataset.

Methods	Two-Level CL			Three-Level CL		
	VAL	ARO	DOM	VAL	ARO	DOM
HA-BGRU [25]	84.6 (10.90)	82.7 (8.25)	84.3 (9.67)	80.2 (8.66)	79.8 (9.60)	80.7 (9.61)
R2G HA-BGRU	88.6 (8.57)	87.1 (8.64)	88.3 (9.95)	84.5 (11.30)	84.4 (10.45)	84.8 (9.48)
R2G TF-Encoder	89.2 (8.07)	87.8 (10.74)	88.9 (8.86)	85.1 (10.32)	84.9 (11.27)	85.4 (10.90)
R2G HSA-BGRU	90.1 (10.22)	88.5 (10.95)	89.5 (9.65)	85.8 (9.89)	85.6 (8.42)	86.1 (11.13)
**HSCFLM**	**93.3** **(9.74)**	**91.6** **(10.71)**	**92.8** **(8.99)**	**88.9** **(10.62)**	**89.1** **(8.89)**	**89.4** **(10.38)**

The standard deviation is provided in parentheses. CL, VAL, ARO, and DOM denote classification, valence, arousal, and dominance, respectively.

**Table 5:** Subject-independent experimental results in four-level classification using the DEAP, MAHNOB-HCI dataset and three-level classification using the SEED dataset.

Methods	Four-Level CL (HAHV vs. LAHV vs. HALV vs. LALV)		Three-Level CL (VAL)
	DEAP	MAHNOB-HCI	SEED
HA-BGRU [25]	74.9 (10.86)	75.2 (8.76)	81.9 (11.59)
R2G HA-BGRU	79.4 (11.03)	79.0 (11.46)	85.8 (12.10)
R2G TF-Encoder	78.9 (8.11)	79.8 (11.83)	86.8 (9.89)
R2G HSA-BGRU	79.5 (10.16)	80.4 (10.34)	87.3 (9.58)
**HSCFLM**	**83.2** **(9.04)**	**83.9** **(9.86)**	**90.9** **(10.15)**

The standard deviation is provided in parentheses. CL and VAL denote classification and valence, respectively.

**Table 6:** Configuration of the proposed model.

Module		Number of Network Parameters	Number of Additions	Number of Multiplications
Regional Brain-Level Encoding	Temporal Encoding	350,976	124,489	13,568,576
	Spatial Encoding	527,616	249,864	627,200
Global Brain-Level Encoding and Classification		639,744	269,097	3,805,440
Total		1,518,336	643,450	18,001,216

**Table 7:** Network parameters, training time, test time per EEG data, network size, and average recognition accuracy of the proposed model.

Model	Network Parameters	Training Time (h:m:s)	Test Time Per EEG Data (ms)	Network Size (MB)	Average Accuracy (%)
HSCFLM	1,518,336	02:59:07	25	14.8	83.2

**Table 8:** Within-subject experimental results in four-level classification using the DEAP and MAHNOB-HCI datasets and three-level classification using the SEED dataset.

Methods	Four-Level CL (HAHV vs. LAHV vs. HALV vs. LALV)				Three-Level CL (VAL)	
	DEAP		MAHNOB-HCI		SEED	
	ACC (STD)	*p*-Value	ACC (STD)	*p*-Value	ACC (STD)	*p*-Value
HA-BGRU [25]	83.6 (9.85)	0.038	83.8 (11.56)	0.031	88.3 (10.06)	0.032
R2G HA-BGRU	88.1 (11.28)	0.016	87.7 (10.37)	0.015	91.9 (12.28)	0.024
R2G TF-Encoder	87.8 (9.20)	0.017	88.5 (9.43)	0.018	92.9 (10.19)	0.017
R2G HSA-BGRU	88.4 (10.79)	0.015	89.3 (11.14)	0.012	93.7 (9.56)	0.014
**HSCFLM**	**92.4 (9.06)**		**93.1 (8.95)**		**97.2 (10.11)**	

CL, VAL, ACC, and STD denote classification, valence, accuracy, and standard deviation, respectively.

**Table 9:** Emotion classification experiment results for each brain region using the proposed model.

Brain Regions	DEAP Two-Level CL		MAHNOB-HCI Two-Level CL		SEED Three-Level CL
	VAL	ARO	VAL	ARO	VAL
*R^1^* (Prefrontal)	86.8	86.5	87.7	86.5	85.4
*R^2^* (Frontal)	86.7	86.1	87.3	86.4	85.2
*R^3^* (Left Temporal)	86.1	85.9	86.5	85.8	85.3
*R^4^* (Right Temporal)	86.2	84.3	86.2	84.8	85.7
*R^5^* (Central)	85.8	85.4	86.9	85.7	84.5
*R^6^* (Left Parietal)	84.1	84.8	84.1	85.4	83.6
*R^7^* (Parietal)	85.7	86.2	85.4	86.2	84.5
*R^8^* (Right Parietal)	85.3	84.6	85.7	86.8	84.3
*R^9^* (Occipital)	83.6	83.1	84.1	83.5	82.7
All Regions	92.1	90.5	93.3	91.6	90.9

CL, VAL, and ARO denote classification, valence, and arousal, respectively.

## Data Availability

The DEAP dataset can be found at https://www.eecs.qmul.ac.uk/mmv/datasets/deap/ (accessed on 26 September 2022). The MAHNOB-HCI dataset is available online at https://mahnob-db.eu/hci-tagging/ (accessed on 7 July 2023). The SEED dataset is available at https://bcmi.sjtu.edu.cn/home/seed/ (accessed on 21 December 2022).
